# Comparison of virucidal activity between weakly acidified chlorous acid water and sodium hypochlorite solution

**DOI:** 10.1099/acmi.0.001033.v5

**Published:** 2026-07-15

**Authors:** Keiko Ikeda, Isanori Horiuchi, Hisataka Goda, Tamiko Nagao, Haruyuki Nakayama-Imaohji, Tomomi Kuwahara, A. Hajime Koyama

**Affiliations:** 1 School of Health and Nursing Science, Wakayama Medical University, Wakayama 641-0011, Japan; 2 Sankei Co. Ltd., 2-2-53, Shiromi, Chuo-ku, Osaka 540-0001, Japan; 3 Department of Science for Human Health Welfare Care Major, Shikoku University Junior College, Ojin-cho, Tokushima 771-1192, Japan; 4 Department of Molecular Microbiology, Faculty of Medicine, Kagawa University Graduate School of Medicine, Kagawa 761-0793, Japan; 5 Wakayama Medical University Graduate School of Medicine, Wakayama 641-0011, Japan

**Keywords:** chlorous acid, disinfectant, sodium hypochlorite, virus inactivation

## Abstract

The virucidal activities of weakly acidified chlorous acid water (WACAW) were characterized in comparison to those of sodium hypochlorite solution (NaClO), using polioviruses type 1 and 3 (PV-1 and PV-3). In the absence of interfering substances, more than 10^−4^ reduction of the infectivity was achieved by NaClO at 5 p.p.m. and by WACAW at 0.75 p.p.m. of free available chlorine (FAC) concentration. Similarly, in the presence of 0.5% polypepton, more than four logs (99.99% or more) reduction of infectious virus for PV-1 and PV-3 was achieved by the incubation with NaClO at 2,000 p.p.m. but not at 500 p.p.m., while it was achieved by the incubation with WACAW at 100 p.p.m. but not at 50 p.p.m. These results show that WACAW was more effective than NaClO at the same FAC concentrations regardless of the presence of interfering substances, suggesting a general advantage of the WACAW for practical applications as a disinfectant.

## Data summary

All data associated with this work are reported within the article.

## Introduction

Chlorine-based solutions have been widely used for disinfection of contaminated surfaces because they have several advantages: they are effective for all microorganisms, environmentally friendly and cheap [1]. The microbicidal mechanism of chlorine is based on oxidative damage to proteins and nucleic acids in microorganisms [2,4]. Among chlorine-based disinfectants, sodium hypochlorite solution (hereinafter referred to as NaClO) is most commonly used but has detrimental effects on tissues due to its alkaline nature, limiting its broader applications [1,5]. In contrast, weakly acidified chlorous acid water (hereinafter referred to as WACAW) is effective as a disinfectant even under acidic conditions and has the advantage of having relatively mild tissue-damaging effects [5]. Considering these advantages, we examined its effectiveness under practical conditions by comparing the virucidal ability of WACAW with that of NaClO in the presence of disinfectant-interfering organic compounds.

Because of the oxidative potential of chlorine-based sanitizers, the effective concentration of chlorine is determined by free available chlorine (FAC) [6,7], not the total chlorine (TC). The FAC concentration is measured using the DPD (*N,N*-diethyl-p-phenylenediamine sulphate) method, while the TC concentration is determined by iodometric titration [8]. In the case of NaClO, the TC level is equivalent to the FAC level, but in the case of WACAW, the TC level is much higher than the FAC level because of the differences in species of chlorinated oxides between these two disinfectants (see the introduction of reference [9]). In this report, we express the concentration of the disinfectants by the FAC concentration, not the TC concentration, because FAC indicates the level of reactive chlorine contributing to the oxidation.

## Methods

Cells and viruses: Vero cells were grown in Eagle’s minimum essential medium (MEM; Shimadzu Diagnostics Corp. #05900) containing 5% FBS. Polioviruses type 1 and 3, which are Sabin live vaccine strains (PV-1 and PV-3, respectively), were used as test viruses. The virus was propagated in Vero cells in MEM supplemented with 0.5% FBS and harvested after three cycles of freezing and thawing of the infected cells along with culture medium [10]. The harvested viruses were centrifuged at 3,500 r.p.m. for 15 min to remove the cell debris and the supernatant fluid was used as a conventional virus stock without further purification and stored at –80 °C. The amount of infectious virus was measured by a plaque assay on Vero cells. The virus concentration of virus stock was 3.1×10^8^ pfu ml^−1^ for PV-1 and 1.0×10^8^ pfu ml^−1^ for PV-3. The calculated ‘limit of detection’ in all experiments in this study for PV-1 was 1/1.6×10^5^ [LRV (log reduction value) is 5.2] and that for PV-3 was 1/0.5×10^5^ (LRV is 4.7).

Reagents: WACAW, an aqueous solution of chlorous acid and NaClO aqueous solution were obtained from Sankei Co. Ltd. (Osaka, Japan). The FAC concentrations of the two solutions were determined just before shipping by the supplier. The FAC concentration of the WACAW used in the experiment is ~1/40 of the TC concentration of the WACAW. Both the original and the diluted solutions were kept in the airtight bottle with minimum air space, stored at 4 °C in the dark and used within a month. During this storage period, no decrease in virus-inactivating activities was observed [5]. As substances interfering with the virus inactivation effects of these reagents, 0.5% polypepton (PP; Shiotani M.S. Co., Ltd. #398-02173) and the mixture of 0.3% sheep red blood cells (SRBC; Japan Bio Serum Co., Ltd. #027-00314-01) and 0.3% BSA (Fujifilm Wako Chemicals, #015-27053) were used throughout the experiments, because these compounds were commonly used as interfering substances in these kinds of study [11]. In addition, we used deionized water as a solvent for the test solution to demonstrate the availability of WACAW as a disinfectant under the practical conditions.

**Fig. 1.**
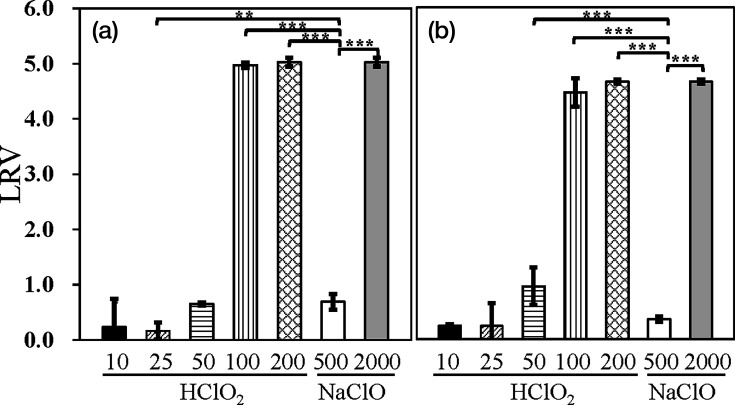
Comparison of two disinfectants to inactivate PV-1 (**a**) and PV-3 (**b**) in the presence of 0.5% PP. The virus was incubated at 25 °C for 5 min with the indicated concentrations of WACAW (black bar, 10 ppm; bar with oblique line, 25 ppm; bar with horizontal line, 50 ppm; bar with vertical line, 100 ppm; bar with cross line, 200 ppm) or NaClO (white bar, 500 ppm; grey bar, 2,000 ppm). After incubation, the number of infectious virus remaining in the samples was determined and normalized to the virus infectivity incubated in the absence of the reagent. Error bars represent two standard deviations (σ) of three independent experiments (*n=3*). Differences in LRV at varying chemical concentrations were statistically compared against the 500 ppm NaClO group using one-way ANOVA followed by Dunnett’s test. Statistical significance is indicated by asterisks (**P<0.01, ***P<0.001).

Assay for virucidal activity: EN14476 Standard method was used to evaluate virucidal activity. To confirm that the disinfectant reacts with the virus in the presence of interfering substances, the virus was mixed first with PP (final 2.5%) or with the mixture of SRBC and BSA (final 1.5% SRBC and 1.5% BSA, respectively) and then allowed to contact with disinfectant preparations. Namely, a 160 µl aliquot of deionized water solutions containing one of the disinfectants at various concentrations received a 40 µl aliquot of the above virus preparations containing interfering substances and were incubated at 25 °C for 5 min. After the incubation, aliquots of these virus samples were immediately diluted 100-fold with ice-cold Dulbecco’s phosphate-buffered saline without Ca^2+^ and Mg^2+^ containing 0.5% FBS and 0.5% PP to prevent further virus inactivation. After the dilution, the concentration of NaClO or WACAW decreased to 20 ppm for NaClO or 2 ppm for WACAW even at the maximum concentration under our experimental condition (shown in Fig. 1), and the presence of 0.5% FBS and 0.5% PP in PBS can completely interfere with the viricidal action of NaClO or WACAW in the diluted samples (5, Ikeda *et al*., unpublished observation). In each experiment, triplicate samples (i.e. three independent test tubes) were prepared for each experimental group. The number of infectious virus in the incubated samples was determined by a plaque assay on Vero cells and was normalized to the average virus infectivity of control samples, incubated in the absence of the reagent. All the experiments were carried out three times or more; at least one experiment was done to determine the dilution factor of each sample necessary to get the proper number of plaques on assay plates and, after the determination of the dilution factors for all samples, we repeated the experiment two times or more until confirming the reproducible results.

Statistical analysis: All data are presented as the mean±standard deviation (σ) derived from three independent experiments. Comparisons between two groups were analysed using Welch’s t-test. For multiple group comparisons, statistical significance was determined using one-way ANOVA followed by Dunnett’s *post-hoc* test. All statistical analyses were computed using the *R* statistical computing environment. A *P*-value of less than 0.05 was considered statistically significant.

## Results and discussion

**Fig. 2.**
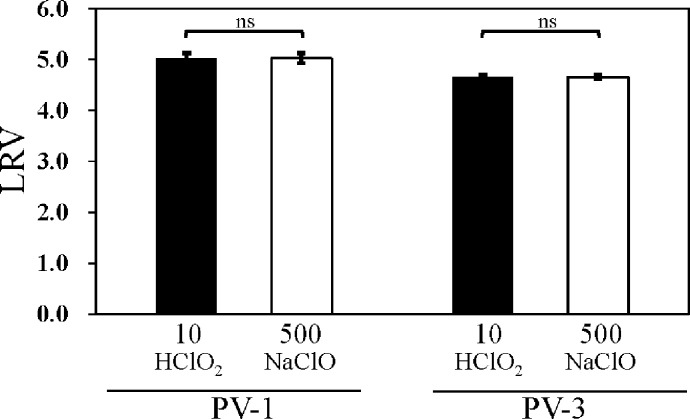
Virucidal activities of WACAW and NaClO against PV-1 and PV-3. The virus was incubated at 25 °C for 5 min with NaClO at 500 ppm (white bar) or WACAW at 10 ppm (black bar). After incubation with each disinfectant, the number of infectious virus remaining in the samples was determined by a plaque assay and normalized to the virus infectivity incubated in the absence of the reagent. Error bars represent two standard deviations (σ) of three independent experiments (*n=3*). No statistically significant difference (ns) was observed between the NaClO and WACAW groups (Welch’s t-test).

Virucidal effect of chlorous acid without interfering substances: For the disinfection of nonenveloped viruses, such as poliovirus and norovirus, it is generally recommended to use a strong disinfectant at high concentrations. For example, ‘Environmental Disinfectant Evaluation Guidelines 2020’ by the ‘Disinfectant Evaluation Committee, Japanese Society for Infection Prevention and Control’ recommends the use of NaClO at 500 ppm (mg l^−1^) for a reliable mode of inactivation of these viruses. As shown in Fig. 2, we confirmed the effectiveness of NaClO at 500 ppm for the complete inactivation of polioviruses under our experimental conditions; i.e. the reagent decreased the infectivity of PV-1 and PV-3 below the detection level (10^−4^ or less) by incubation at 25 °C for 5 min in deionized water without any interfering substances, such as organic compounds (white bar in the figure). When we examined the ability of WACAW to inactivate PV-1 or PV-3 under the same experimental conditions, WACAW at 10 ppm of FAC concentration could reduce the infection titre of PV-1 and PV-3 similarly to NaClO (black bar in Fig. 2). It should be noted that 10 ppm FAC concentration of WACAW, determined by the DPD method, is equivalent to 400 ppm of TC concentration, while FAC and TC concentrations are the same for NaClO.

**Fig. 3.**
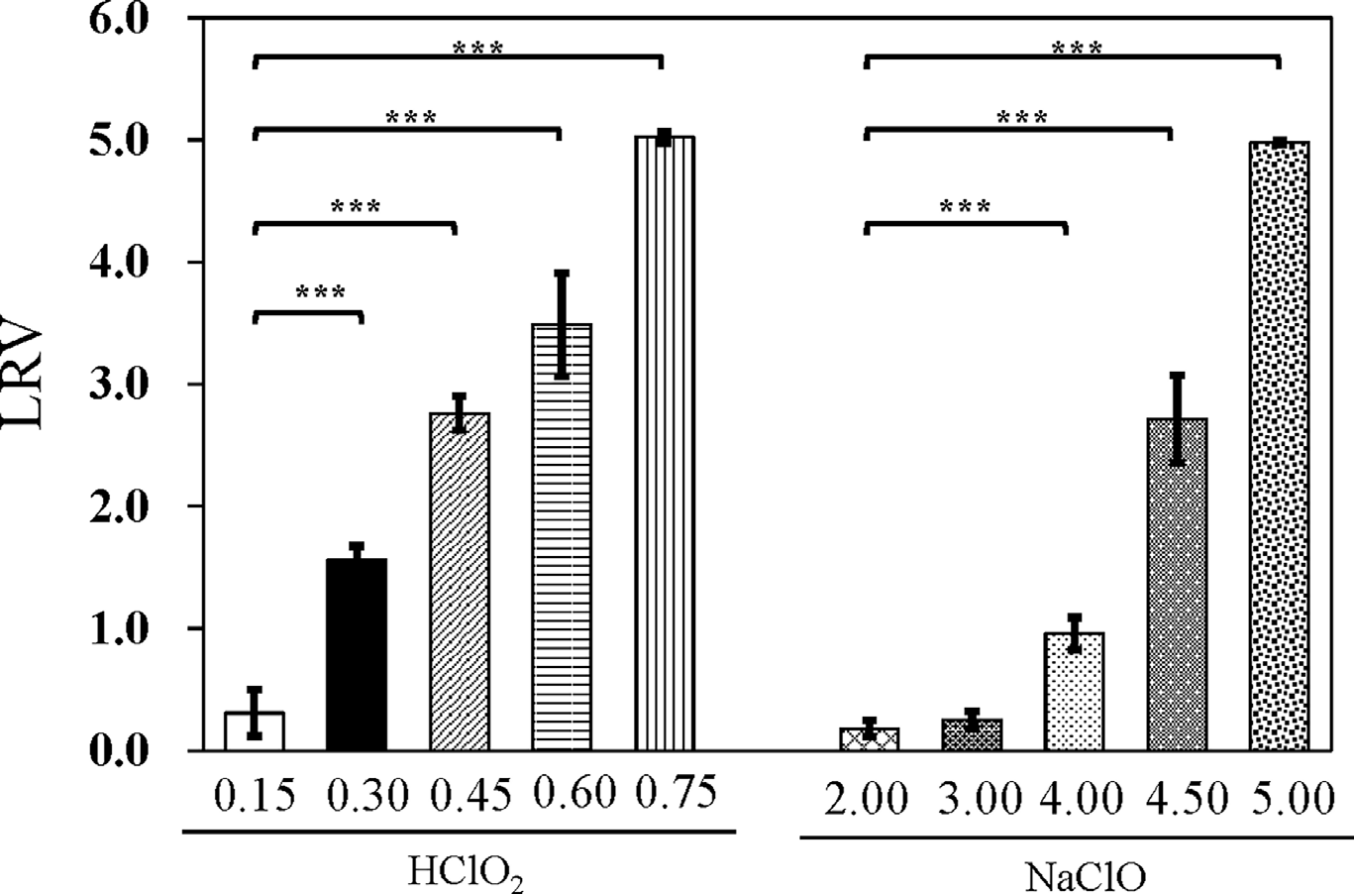
Comparison of the ability of two disinfectants to inactivate PV-1. The virus was incubated at 25 °C for 5 min with the indicated concentrations of NaClO or WACAW. After incubation, the number of infectious virus remaining in the samples was determined and normalized to the virus infectivity incubated in the absence of the reagent. Error bars represent two standard deviations (σ) of three independent experiments (*n=3*). Differences in LRV at varying chemical concentrations were statistically compared with the lowest concentration using one-way ANOVA followed by Dunnett’s test. Statistical significance is indicated by asterisks (***P<0.001).

To further characterize the virucidal activity of WACAW, we compared the concentration-dependency of virucidal effects of WACAW and NaClO on PV-1. As shown in Fig. 3, more than 10^−4^ reduction of the infectivity was achieved by WACAW at 0.75 ppm and the similar reduction was achieved by NaClO at 5 ppm, indicating greater effectiveness of WACAW. When PV-1 was incubated with various concentrations of these disinfectants at 25 °C for 5 min, the number of infectious viruses started decreasing at 0.3 ppm and gradually reached the detection limit at 0.75 ppm, while with NaClO, the number started decreasing at 4 ppm and rapidly reached below detection level at 5 ppm, confirming the effectiveness of WACAW as a virucidal agent; i.e. WACAW was sevenfold (5 ppm for NaClO/0.75 ppm for WACAW) more effective than NaClO on PV-1 to achieve the detection level under the conditions without interfering substances.

**Fig. 4.**
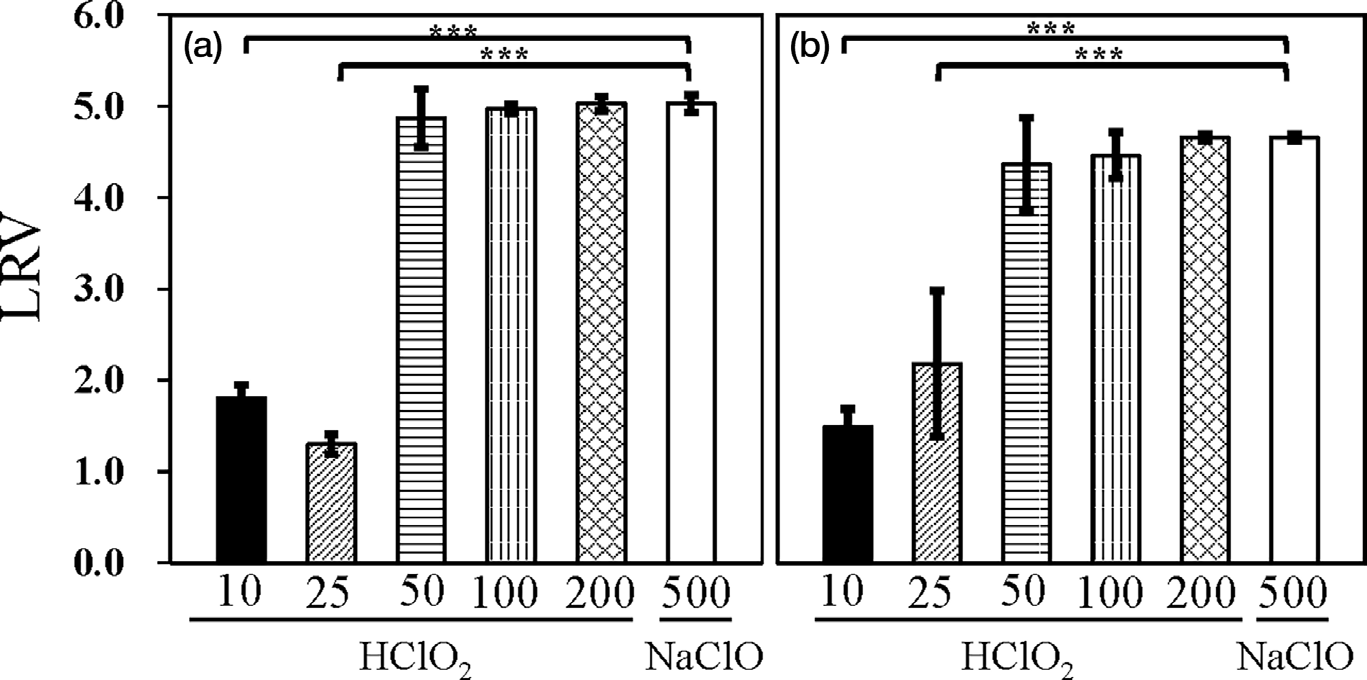
Comparison of the ability of two disinfectants to inactivate PV-1 (**a**) and PV-3 (**b**) in the presence of 0.3% BSA and 0.3% SRBC. The virus was incubated at 25 °C for 5 min with the indicated concentrations of WACAW (black bar, 10 ppm; bar with oblique line, 25 ppm; bar with horizontal line, 50 ppm; bar with vertical line, 100 ppm; bar with cross line, 200 ppm) or NaClO (white bar, 500 ppm). After incubation, the number of infectious virus remaining in the samples was determined and normalized to the virus infectivity incubated in the absence of the reagent. Error bars represent two standard deviations (σ) of three independent experiments (*n=3*). Differences in LRV at varying chemical concentrations were statistically compared against the 500 ppm NaClO group using one-way ANOVA followed by Dunnett’s test. Statistical significance is indicated by asterisks (***P<0.001).

Virucidal effect of WACAW in the presence of inhibitory organic substances: To compare the virucidal ability of WACAW with that of NaClO in the practical environment, we examined their activity in the presence of inhibitory organic substances. As shown in Fig. 4, when the ability of WACAW or NaClO to inactivate PV-1 and PV-3 in the presence of 0.3% BSA and 0.3% SRBC was determined, more than four logs (99.99% or more) reduction of infectious virus for PV-1 (Fig. 4a) and PV-3 (Fig. 4b) was achieved by the incubation at 25 °C for 5 min with NaClO at 500 ppm or by that with WACAW at 50 ppm or higher, confirming the effectiveness of WACAW as a practical disinfectant under these conditions.

To examine the effect of differences in the interfering substance (i.e. contaminating organic compounds), we used 0.5% PP instead of a mixture of 0.3% BSA and 0.3% SRBC. When 0.5% PP was used, similar results were obtained, although the inhibitory effect of PP was stronger than the mixture of BSA and SRBC. As shown in Fig. 1, more than four logs (99.99% or more) reduction of infectious virus for PV-1 (Fig. 1a) and PV-3 (Fig. 1b) was achieved by the incubation with NaClO at 2,000 ppm, but not at 500 ppm WACAW at 100 or 200 ppm, but not below 50 ppm, resulted in more than four logs reduction of infectious virus for both PV-1 (Fig. 1a) and PV-3 (Fig. 1b). These results also show that WACAW can effectively inactivate PV-1 and PV-3 even in the presence of 0.5% PP and that WACAW is significantly more effective than NaClO based on the concentration unit of FAC.

## Conclusion

We have characterized the virucidal activities of WACAW against various viruses [5,9, 12, 13]. In this study, we focus on the activity in the practical environment and on non-enveloped viruses. Non-enveloped viruses, such as polioviruses, are generally known to be more resistant to both chemical and physical treatments than enveloped viruses and, therefore, are good indicators to evaluate the virucidal abilities of disinfectants. Comparing with the virucidal activity of NaClO, an authentic widely used disinfectant, the results in this paper show that WACAW was more effective than NaClO if compared on the basis of the FAC concentration of the two reagents and also sufficiently effective if compared by the TC concentration of the reagents. The tissue-friendly nature of WACAW [5], in addition to the observed marked virucidal activity, would provide a basis for the use of WACAW as a disinfectant in food hygiene and as a sanitizer in the health care fields, such as a health care facility for the elderly and a daycare/preschool.

## Abbreviations

DPD: N,N diethyl-p-phenylenediamine sulfate
FAC: free available chlorine
LRV: log reduction value
MEM: minimum essential medium
NaClO: sodium hypochlorite solution
PP: polypepton
PV-1: poliovirus type 1
PV-3: poliovirus type 3
SRBC: sheep red blood cells
TC: total chlorine
WACAW: weakly acidified chlorous acid water

## References

[1] Rutala WA, Weber DJ (1997). Uses of inorganic hypochlorite (bleach) in health-care facilities. *Clin Microbiol Rev*.

[2] Haas CN, Engelbrecht RS (1980). Physiological alterations of vegetative microorganisms resulting form chlorination. J Water Pollut Control Fed.

[3] Young SB, Setlow P (2003). Mechanisms of killing of Bacillus subtilis spores by hypochlorite and chlorine dioxide. J Appl Microbiol.

[4] Van Aken B, Lin L-S (2011). Effect of the disinfection agents chlorine, UV irradiation, silver ions, and TiO2 nanoparticles/near-UV on DNA molecules. Water Sci Technol.

[5] Goda H, Ikeda K, Nishide M, Nagao T, Koyama AH (2018). Characterization of virucidal activities of chlorous acid. Jpn J Infect Dis.

[6] Butterfield CT (1948). Bactericidal properties of free and combined available chlorine. *J AWWA*.

[7] Ridenour GM, Ingols RS (1946). Inactivation of poliomyelitis virus by “free” chlorine. Am J Public Health Nations Health.

[8] Ingram PR, Pitt AR, Wilson CG, Olejnik O, Spickett CM (2004). A comparison of the effects of ocular preservatives on mammalian and microbial ATP and glutathione levels. Free Radic Res.

[9] Goda H, Yamaoka H, Nakayama-Imaohji H, Kawata H, Horiuchi I (2017). Microbicidal effects of weakly acidified chlorous acid water against feline calicivirus and *Clostridium difficile* spores under protein-rich conditions. PLoS One.

[10] Koyama AH, Irie H, Ueno F, Ogawa M, Nomoto A (2001). Suppression of apoptotic and necrotic cell death by poliovirus. J Gen Virol.

[11] Disinfectant Evaluation Committee, Japan Society for Environmental Infections (2020). Environmental disinfectant evaluation guidelines.

[12] Horiuchi I, Kawata H, Nagao T, Imaohji H, Murakami K (2015). Antimicrobial activity and stability of weakly acidified chlorous acid water. Biocontrol Sci.

[13] Yamaoka H, Nakayama-Imaohji H, Horiuchi I, Yamasaki H, Nagao T (2016). Tetramethylbenzidine method for monitoring the free available chlorine and microbicidal activity of chlorite-based sanitizers under organic-matter-rich environments. Lett Appl Microbiol.

